# An audiological profile of patients infected with multi-drug resistant tuberculosis at a district hospital in KwaZulu-Natal

**DOI:** 10.4102/sajcd.v63i1.154

**Published:** 2016-11-25

**Authors:** Delicia Appana, Lavanithum Joseph, Jessica Paken

**Affiliations:** 1 Department of Health, Durban, South Africa; 2 Discipline of Audiology, University of KwaZulu-Natal, South Africa

## Abstract

**Background:**

The increased incidence of multi-drug-resistant tuberculosis (MDR-TB) and the consequent use of aminoglycosides with their ototoxic potential necessitate a better understanding of the audiological pattern of infected patients.

**Objective:**

To describe the occurrence and nature of hearing loss in patients with MDR-TB receiving aminoglycosides over a period of 6 months.

**Methods:**

Baseline and five consecutive monthly audiological assessments were conducted on 52 adults at a hospital in KwaZulu-Natal. A longitudinal descriptive study was implemented. A conventional audiological test battery, extended high frequency audiometry and otoacoustic emission testing were conducted. Data were analysed using SPSS version 19 statistical software package.

**Results:**

Decreased hearing was the most common audiological symptom experienced. Bilateral sensorineural hearing loss was predominant. Ototoxic hearing loss was noted in 27 participants (52%) in 1 month post-treatment. Hearing loss progressed from mild to moderate at post-treatment one, to moderate to severe at post-treatment three and severe to profound at post-treatment five. Changes in hearing function were noted in 52 participants (100%) by post-treatment five. High and ultra-high frequencies were most affected. Speech discrimination scores deteriorated over time. The number of patients with absent distortion product otoacoustic emissions increased over treatment duration.

**Conclusion:**

The greatest effects were observed in the high frequencies before manifesting in the lower frequencies. This highlights the importance of inclusion of high frequency audiometry in the early detection of ototoxicity which can go undiagnosed with traditional audiometry. The high prevalence of hearing loss has implications for the provision of audiological service to this patient population.

## Introduction

South Africa is in the midst of a tuberculosis (TB) epidemic and is ranked fourth out of the 22 high-burden countries identified worldwide in terms of TB by the World Health Organization (WHO) (2008). According to the 2011 statistics of the estimated 10 819 139 people living in KwaZulu-Natal, the prevalence of TB was 400 per 100 000, with an incidence of 490 per 100 000 population (WHO, 2011). Whilst these statistics are high, the actual burden of tuberculosis may yet be underestimated in this region (Mitnick, Appelton & Shin, 2008). With the increase in the number of TB patients entering the health care system, it is crucial for health care professionals to be aware of this disease. The high incidence and prevalence of TB infection can be partially attributed to the easy spread of the disease. Further, the emergence of multi-drug-resistant tuberculosis (MDR-TB) associated with antibiotic resistance poses a barrier to the ongoing struggle of global TB control and is subsequently a major challenge to health care needs within South Africa (Zager & McNerney, 2008).

MDR-TB refers to the strains of TB resistant to the two main anti-TB drugs, namely isoniazid and rifampicin (Zager & McNerney, 2008). As part of the WHO recommended guidelines for the effective treatment of MDR-TB, infected patients receive prolonged treatment with category IV treatment regimens that include the use of second-line aminoglycosides such as Kanamycin, Amikacin, Capreomycin, Streptomycin and Viomycin (WHO, 2008). However, the foremost disadvantage of aminoglycoside use is the resultant adverse effects on an individual’s auditory, vestibular and renal function (Peloquin *et al*., 2004). Therefore, the development of MDR-TB has direct implications for audiological assessment and management of infected patients, creating a need to develop monitoring protocols and document the audiological effects of medication in patients infected with MDR-TB. Hence, it is important for audiologists to be actively involved in the multi-disciplinary team management of such patients.

The occurrence and nature of hearing loss in patients infected with MDR-TB is not well documented. Patients receiving aminoglycosides for the treatment of MDR-TB experience disruption of cochlear activity, which results in ‘aminoglycoside-induced ototoxicity’ (Guthrie, 2008, p. 91). Hearing loss sustained owing to ototoxicity may be influenced by patient susceptibility as well as factors related to accumulative effects of the drug over time. Incidence of hearing loss associated with aminoglycosides is reported to be time- and dose-dependent, related to drug half-life and concentrations in the inner ear (Schact, Talaska & Rybak, 2012).

The effects of ototoxicity are initially seen in the outer hair cells at the basal end of the cochlea, with the reception of high frequency sounds therefore being affected first (Duggal & Sarkar, 2007; Guthrie, 2008; Li & Steyger, 2009). Thereafter, there is progressive damage towards the apical end of the cochlea, resulting in the reception of low-frequency sounds being affected. From this, it is understood that with time hearing of the entire speech frequency range will deteriorate (Jacob, Aguiar, Tomiasi, Tschoeke & de Bitencourt, 2006). This, in turn, may directly impact on the patient’s communication and quality of life. Therefore, even at the onset of ototoxicity when the reception of ultra-high frequencies (10.0 kHz and 12.5 kHz) is affected, monitoring hearing loss becomes important.

Considering the highly infectious nature of TB and the ongoing challenge of MDR-TB, it is crucial for health care professionals, including audiologists, to be involved in the management of the patient infected with MDR-TB. Early identification of hearing loss in such patients is a vital responsibility of the audiologist in order to reduce the negative impact of the hearing loss. It may be that treatment regimens are altered from conventional multiple daily doses to a once daily dose in order to reduce the ototoxic effects of aminoglycosides (Barclay, Kirkpatrick & Begg, 1999; Duggal & Sakar, 2007). This then will result in enhanced efforts to slow down the progression towards permanent hearing loss, before those frequencies responsible for speech are affected (Stavroulaki *et al*., 1999) and the hearing loss influences the individual’s quality of life.

The sense of hearing can greatly influence an individual’s quality of life. Hearing loss in adults may reduce communication ability with spoken language, and participation in social and vocational activities, which subsequently impacts on the quality of life of the hearing impaired individual (Boothroyd, 2007). An important goal of the audiologist is to improve a patient’s quality of life by enhancing communication abilities (American Academy of Audiology [AAA], 2009). Quality of life is a global imperative, regardless of whether the patient has a life-threatening disease or not. In an effort to reduce the negative impacts of hearing loss, audiologists play an important role in the care of patients infected with TB (Khoza-Shangase, Mupawose & Mlangeni, 2009). Thus, it is necessary for audiologists to better understand the audiological patterns of patients on TB treatment regimens. Thus, the study aimed to describe the audiological profiles of patients on medication for MDR-TB, over a 6 month period, corresponding to the initial intensive phase of MDR-TB treatment.

## Methods

### Objective

The objective of this study was to describe the occurrence and nature of hearing loss in terms of type and degree of hearing loss in patients with MDR-TB receiving aminoglycoside treatment over a period of 6 months

### Study design

A quantitative longitudinal research design was used. A panel study was used as an investigation was conducted on the same set of participants over the study duration to obtain accurate data changes over time (Rubin & Babbie, 2009).

### Research context

The study was conducted at a district hospital in KwaZulu-Natal which receives referrals from eight satellite clinics in this area. It has an MDR-TB unit which caters for 60 in-patients receiving direct observation treatment. The hospital was purposefully selected because of to its large referral base.

### Participants

#### Sampling technique

A purposive sampling technique was used to recruit all participants commencing the 6-month intensive MDR-TB treatment phase during the study period.

The following participant selection criteria were adhered to:

- All participants older than age 18 were included irrespective of risk factors for hearing loss owing to the benefit of repeated measures used in the study. Furthermore, children were excluded owing to the sensitivities around informed consent for this population as well as improved reliability during subjective assessments. None of the participants fell into the range for presbycusis.
- Participants had to be admitted as in-patients at the hospital with a positive MDR-TB diagnosis.

#### Description of participants

The study sample consisted of 52 patients, of which 27 (52%) were males and 25 (48%) were females. The age range of participants was 18–56 years, with a mean age of 34 years. All participants were black South Africans with 42 (81%) isiZulu first language speakers. Educational level ranged from grade 7 to grade 12.

None of the 52 participants had previous history of aminoglycoside treatment. Forty-nine participants (94%) were HIV-positive, whereas seven participants (14%) had previous occupational noise exposure. In addition, one participant had previous surgery to the head or neck and another participant had a family history of hearing loss.

**FIGURE 1:**
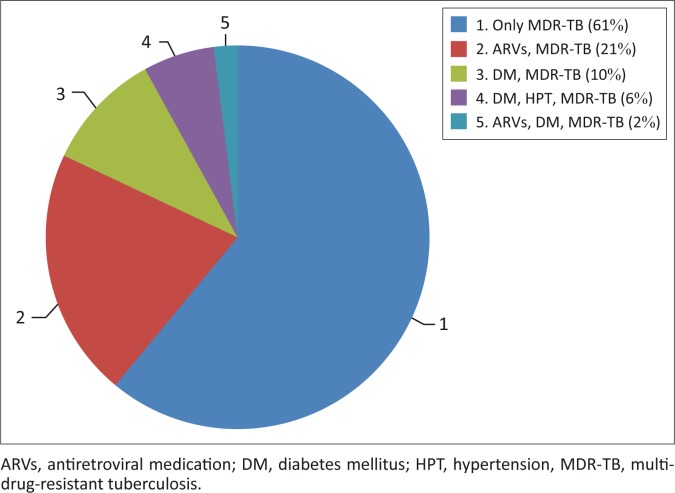
Treatment of other co-morbidities.

All participants (100%) were receiving Kanamycin for the treatment of MDR-TB. Thirty-two participants (61%) were only receiving Kanamycin whilst 11 (21%) were receiving both Kanamycin and antiretroviral medication. Further information on other co-existing conditions is reflected in Figure 1.

### Data collection

Baseline audiological assessments were conducted on each patient within 24 hours of receiving MDR-TB treatment. All procedures were conducted by the research audiologist, who had basic and clinical isiZulu conversational skills developed at university foundation level. The following audiological test battery was used:

- case history information
- otoscopic examination
- immittance audiometry, including tympanometry and ipsilateral acoustic reflex threshold testing at 0.5, 1, 2 and 4 KHz
- pure tone audiometry, including high frequencies of 10 kHz and 12.5 kHz
- speech audiometry, including speech reception threshold and speech discrimination threshold testing using monitored live voice testing and IsiZulu word lists commonly used in hospitals in KwaZulu-Natal
- distortion product otoacoustic emissions (DPOAE).

Audiological equipment used included the Heine Mini 2000 handheld otoscope, GSI 38 Auto-Tymp Immittance meter, Bio-logic Audx Diagnostic Otoacoustic Emission Machine, Madsen Orbiter 922 twin channel Clinical Audiometer (Version 2) with TDH-39 headphones and a single suite sound proof audiological testing booth.

Participants were required to undergo the same tests as the baseline audiological assessment, on a monthly basis for the next 5 months, prior to their next dose of medication. The study broadly used the 1994 ASHA Guidelines (AAA, 2009) in terms of the audiological test procedures, except for the sensitivity range of ototoxicity for threshold determination in order to meet the study objective of describing a detailed audiological profile of patients. The timelines for audiological monitoring assessments were in keeping with the current practice at the study site owing to resource, time and personnel constraints. Additional data from follow-up case history interviews and review of medical records served to supplement audiological information.

### Data analysis

Data were analysed using descriptive and inferential statistics. Information from the initial and follow-up case history questionnaire, medical history review and otoscopic examination checklist were coded and entered onto the SPSS version 19 statistical software package (IBM Corp, 2010). Detailed responses to open-ended questions were analysed using thematic analysis (Neuman, 1997). Results were described with reference to percentiles and medians as data obtained were not normally distributed (Leedy & Ormrod, 2005). Data from all test procedures were analysed using the cross-check principle and described in Table 1.

**TABLE 1:** Audiological assessment and statistical procedures for analysis.

Audiological assessment	Statistical procedure
Case history	Frequencies and percentages were calculated for categorical responses.
Medical history review	Frequencies and percentages were calculated for categorical responses.
Otoscopic examination	Frequencies and percentages were calculated for categorical responses. McNemar tests were used to determine the relationship between case history reports of pain and perforated tympanic membrane observed on otoscopy. Thus, the cross-check principle was ap-plied.
Immittance audiometry	Frequencies and percentages were calculated for the results of tympanograms and acoustic reflex threshold testing.
Pure tone audiometry	Non-parametric statistics were used to analyse thresholds averaged from pure tone audiometry as they were not normally distributed (Leedy & Ormrod, 2005). Friedman’s test was used to analyse change in low-, mid-, high- and ultra-high frequencies from baseline to post-treatment five. In addition, change from one post-treatment session to the next was described using the median and interquartile ranges.
Speech audiometry	Frequencies and percentages were calculated for results of speech audiometry.
Distortion product otoacoustic emissions.	The frequency and percentage of pass and/or fail overall re-sult was determined. In addition, linear regression was used to determine the relationship between DPOAEs and reports of tinnitus.

*Source*: Glattke, T.J., & Robinette, M.S. (2007). Otoacoustic emissions. In R.J. Roeser, M. Valente, & H. Hosford-Dunn (Eds.), *Audiology diagnosis* (2nd edn., pp. 478–496). New York: Thieme Medical Publishers, Inc; Biologic Systems Corp. (2005). Evoked Otoacoustic Emission Measurement System. User’s Manual. Illinois: Biologic Systems Corp.

Note: DPOAE’s were measured across the frequency range 750 Hz – 8000 Hz using the Vanderbilt 65/55 2 St. Dev’ norms and an f2/f1 ratio of 1: 1.22. According to Glattke & Robinette (2007), there are no universally accepted normative values for DPOAE’s thus DPOAE and noise floor levels were analysed. Thereafter, an overall failed DPOAE result was classified as 70% of failed frequencies for the purpose of the study (Biologic Systems Co-operation, 2005).

Changes in hearing function from baseline to monitoring sessions were determined using ASHA (1994) criteria for clinically significant hearing change for early detection and monitoring of ototoxicity (Fausti, Wilmington, Helt, Helt & Konrad-Martin, 2005). Thus, the criteria used were as follows: (1) >20 dB pure tone threshold shift at one frequency, (2) >10 dB shift at two consecutive frequencies, and (3) threshold response shifting to ‘no response’ at three consecutive test frequencies. Pure tone audiometric results were averaged and grouped into four frequency categories (Rademaker-Lakhai *et al*., 2006) as evident in Table 2.

**TABLE 2:** Averaged low-, mid-, high- and ultra-high frequency groups.

Group	Category	Frequencies averaged (Hz)
Group 1	Low-frequency average	125, 250 and 500
Group 2	Mid-frequency average	1000 and 2000
Group 3	High frequency average	4000 and 8000
Group 4	Ultra-high frequency average	10 000 and 12 500

Speech discrimination scores were classified as excellent, good, fair, poor, very poor and extremely poor (Hodgson, 1980).

## Ethical considerations

Ethical clearance was obtained from the University of KwaZulu-Natal Biomedical Research and Ethics Committee (BE253/010). Permission was obtained from the KwaZulu-Natal Department of Health and the medical manager at the study site. All participants were informed of the nature of the study and provided consent to participate in the study. Information sheets and consent forms were available in English and isiZulu. Participants were informed that there will be no names included in the study, thereby maintaining anonymity. In addition, participants were informed that the procedures were not invasive, were commonly used and therefore not harmful and that they were free to withdraw from the study at any point without repercussions for services at the hospital. As per the protocol at the study site, all patients identified with hearing loss received intervention in the form of counselling, hearing aid assessment and fitting following placement on a waiting list for hearing aids, communication strategies training and management for tinnitus.

### Reliability and validity

A battery of audiological test procedures and the cross-check principle were used to enhance reliability and validity. Normative data used to analyse the audiological test battery came from evidence-based research and were compared with each other. Subjective data were compared with objective data during audiological testing. A pilot study was conducted. All equipment was calibrated by a qualified technician prior to data collection and daily biological calibration was conducted by the researcher.

## Results

### Case history information

At baseline, 44 participants (85%) reported no audiological problems. However, reports of hearing loss increased with each successive assessment over treatment duration. At baseline, only four participants (8%) reported hearing loss; however, by post-treatment five, 44 participants (85%) reported decreased hearing, as evident in Table 3. The most common complaint was difficulty hearing in group situations and during administration of medication. Vertigo was reported by a small number (up to 8%) but resolved over time. Reports of tinnitus increased (up to 21%) but dropped to 12%.

**TABLE 3:** Relevant case history findings over treatment duration.

Symptoms	Baseline (%)	PT1 (%)	PT2 (%)	PT3 (%)	PT4 (%)	PT5 (%)
No problems	85	52	46	46	29	14
Pain	6	6	4	0	0	0
Discharge	2	6	6	4	0	0
Blocked ears	10	15	13	17	15	10
Decreased hearing	8	19	40	52	65	85
Tinnitus	2	19	19	21	17	12
Vertigo	0	4	4	8	2	0
Other	0	0	4	6	2	0
General change in health	0	25	25	31	27	25
Change in medication dosage	0	0	8	25	33	37

PT1, post-treatment 1; PT2, post-treatment 2; PT3, post-treatment 3; PT4, post-treatment 4; PT5, post-treatment 5.

### Otoscopic examination and immittance audiometry

Type A tympanograms were most common over treatment duration in 49 participants (94%) with clear ear canals and intact tympanic membranes on otoscopy. Two participants (4%) had tympanic membrane perforations, one (2%) bilaterally and one (2%) on the right tympanic membrane. As treatment duration progressed, the number of participants with absent or reduced acoustic reflex thresholds increased, and sensation levels were reduced.

### Pure tone audiometry

#### Occurrence of hearing loss

**FIGURE 2:**
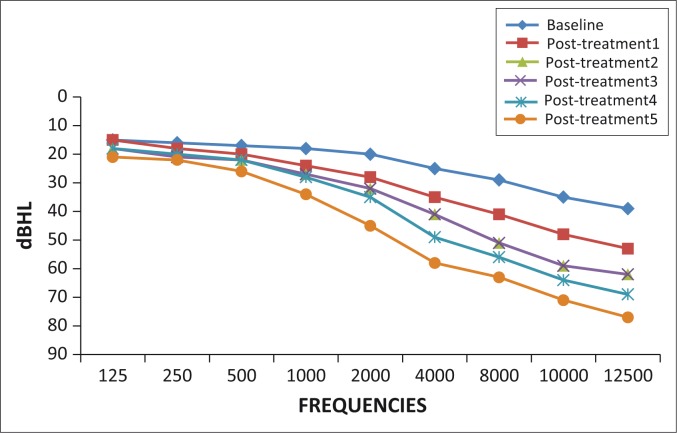
Pure tone audiometry mean thresholds over treatment duration: Right ear.

**FIGURE 3:**
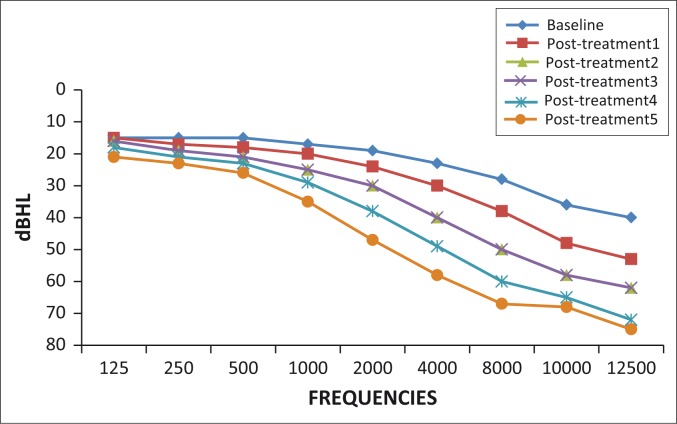
Pure tone audiometry mean thresholds over treatment duration: Left ear.

All participants (100%) showed changes for one or more of the ASHA (1994) criteria for clinically significant hearing change from baseline to post-treatment five. At baseline, normal hearing was observed in 23 participants (44%) on the right ear and 21 participants (40%) on the left ear. By post-treatment five, all 52 participants (100%) showed changes for one or more of the ASHA (1994) criteria for clinically significant hearing change from baseline to post-treatment five bilaterally. Changes in hearing function with pure tone audiometry are highlighted in Figures 2 and 3.

#### Description of hearing loss

Hearing thresholds from baseline to post-treatment five were described according to four frequency averages highlighted in Table 2.

**FIGURE 4:**
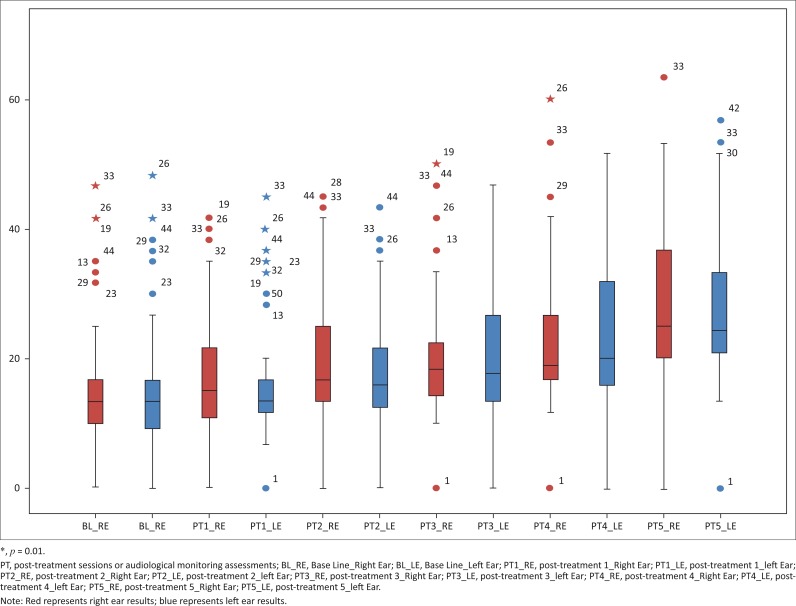
Low-frequency threshold changes over treatment duration.

**Low frequencies:** The low-frequency median on the left ear was 13.33 (IQR 10.00–17.08) at baseline. This value deteriorated to 25.00 (IQR 20.00–37.08) at post-treatment five. A similar trend was noted on the right ear with low frequencies. The median at baseline was 13.33 (IQR 9.58–17.08) and 23.33 (IQR 21.25–33.33) at post-treatment five. Gradual deterioration of low-frequency medians were noted over each consecutive post-treatment assessment; however, the averages remained within normal limits. See Figure 4.

**FIGURE 5:**
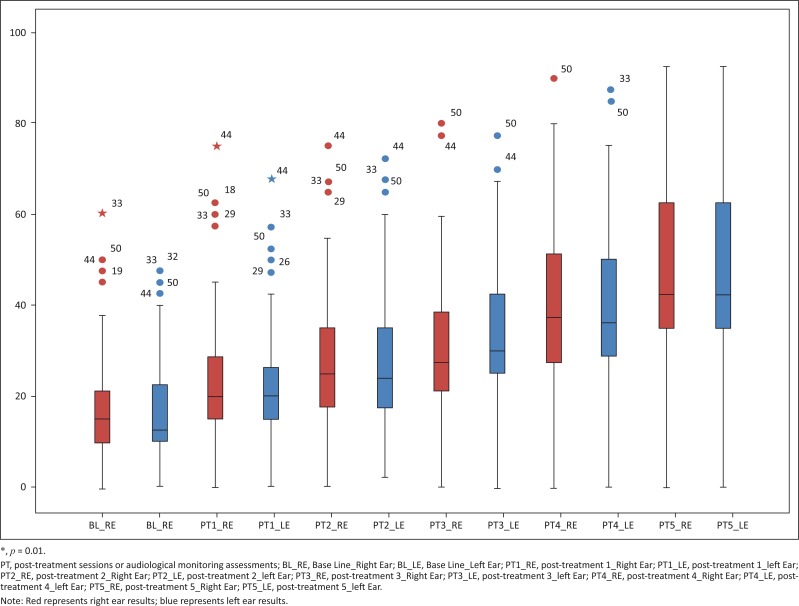
Mid-frequency threshold changes over treatment duration.

**Mid-frequencies:** On the right ear, pure tone mid-frequency medians were 15.00 (IQR 10.00–22.50) at baseline. At post-treatment five, deterioration to 42.50 (IQR 36.88–62.50) was observed. Similarly, on the left ear, mid-frequency pure tone medians at baseline were 5.00 (IQR 10.00–22.50) and 42.50 (IQR 36.88–62.50) at post-treatment five. Thus, there was an increase in pure tone mid-frequency thresholds from baseline to post-treatment five (see Figure 5).

**FIGURE 6:**
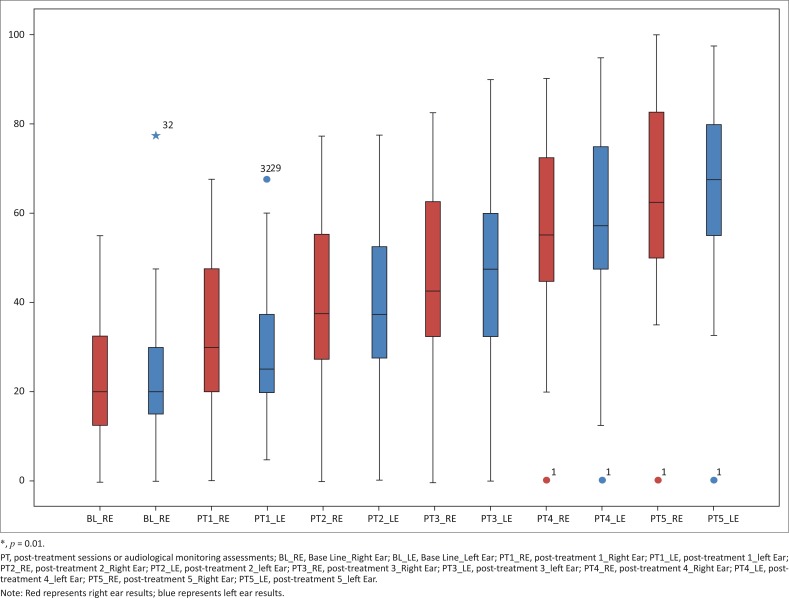
High frequency threshold changes over treatment duration.

**High frequencies:** The high frequency median at baseline was 20.00 for both right (IQR 15.00–33.75) and left (15.00–32.50) ears. At post-treatment five, this value increased to 65.00 (IQR 51.25–85.00) on the right ear and 70.00 (IQR 57.50–85.00) on the left ear. At each consecutive post-treatment assessment, the high frequency medians increased, thereby suggesting gradual deterioration each month (see Figure 6).

**FIGURE 7:**
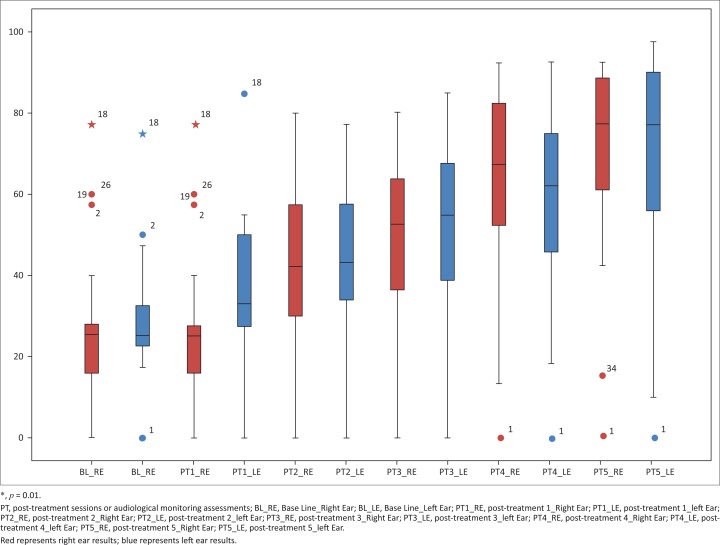
Ultra-high frequency threshold changes over treatment duration.

**Ultra-high frequencies:** On the right ear, the ultra-high frequency median was 25.00 (IQR 20.00–35.00) at baseline. At post-treatment five, marked deterioration to 82.50 (IQR 70.00–92.50) was observed. Similarly, on the left ear, ultra-high frequency median at baseline was 25.00 (IQR 22.50–38.13) which increased to 87.50 (IQR 63.75–95.00) at post-treatment five. Therefore, the ultra-high frequencies were the most affected during pure tone audiometry (see Figure 7).

Hearing loss was most prominent in the ultra-high frequencies initially. However, over the five post-treatment assessments, high, mid and low frequencies were gradually affected. The degree of hearing loss was initially mild to moderate at post-treatment one and gradually deteriorated to severe to profound by post-treatment five.

**FIGURE 8:**
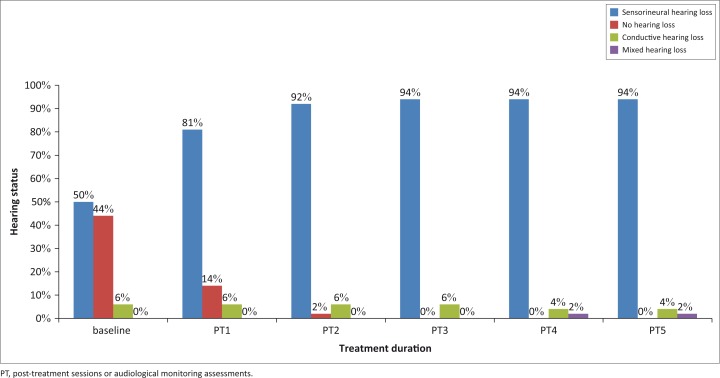
Type of hearing loss over treatment duration (right ear).

**FIGURE 9:**
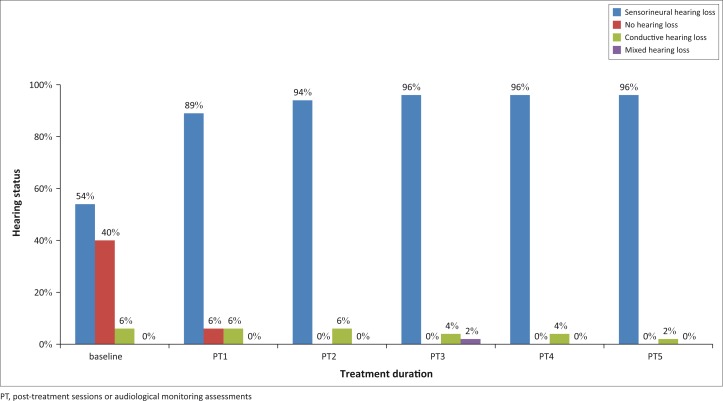
Type of hearing loss over treatment duration (left ear).

At post-treatment one, sensorineural hearing loss was most common in 42 participants (81%) on the right ear (Figure 8), and 46 participants (89%) on the left ear (Figure 9). The occurrence of sensorineural hearing loss continued to be most common on subsequent audiological assessments, as evident in Figure 4 and Figure 5.

### Speech audiometry

At baseline, 48 participants (92%) presented with good speech discrimination and four (8%) presented with fair speech discrimination. At post-treatment five, speech audiometry was conducted on only 27 participants due to participant reports of fatigue or speech distortion. Thus, participants were unable to cope with speech audiometry as treatment duration progressed. Of these 27 participants, 6 participants (22%) presented with very poor speech discrimination scores, as reflected in Table 4.

**TABLE 4:** Speech discrimination results: Speech discrimination (Better ear).

Norms (Hodgson, 1980)	Baseline†		PT1†		PT2‡		PT3§		PT4¶		PT5¶	
					
	*n*	%	*n*	%	*n*	%	*n*	%	*n*	%	*n*	%
Excellent (90% – 100%)	0	0	0	0	0	0	0	0	0	0	0	0
Good (82% – 90%)	48	92	48	92	21	55	19	66	21	66	21	78
Fair (72% – 80%)	4	8	4	8	11	29	0	0	0	0	0	0
Poor (52% – 70%)	0	0	0	0	5	13	8	27	9	28	0	0
Very poor (22% – 50%)	0	0	0	0	1	3	2	7	2	6	6	22
Extremely poor (<20%)	0	0	0	0	0	0	0	0	0	0	0	0

† *n* = 52

‡ *n* = 38

§ *n* = 29

¶ *n* = 32

†† *n* = 27

PT1, post-treatment 1; PT2, post-treatment 2; PT3, post-treatment 3; PT4, post-treatment 4; PT5, post-treatment 5.

### Distortion production otoacoustic emissions

**FIGURE 10:**
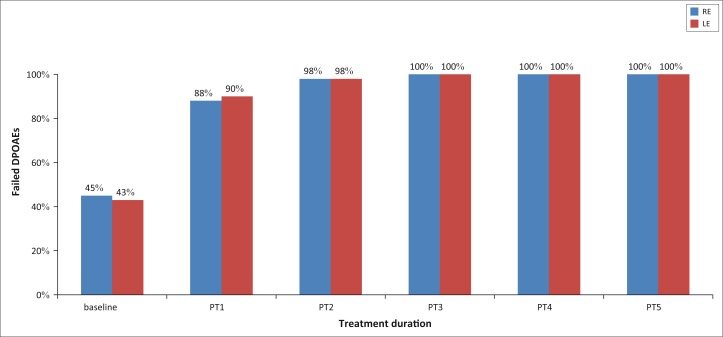
Distortion product otoacoustic emissions over treatment duration.

The number of participants with absent DPOAEs increased over treatment duration. At baseline, 27 participants (55%) presented with absent DPOAEs on the right ear and 22 participants (45%) on the left ear. By post-treatment five, all 52 participants (100%) presented with absent DPOAEs bilaterally as reflected in Figure 10.

## Discussion

The auditory symptoms reported by participants in this study are in keeping with symptoms of ototoxicity. Case history interviews conducted at baseline and post-treatment assessments revealed increased reports and then a plateau in the number of participants who complained of tinnitus. Tinnitus is commonly associated with cochlear hearing loss, associated with aminoglycosides, such as in patients on treatment for MDR-TB. The reports of vertigo could be associated with Kanamycin which is reported to have more cochlear than vestibular effects. However, vertigo may have been confused with weakness or fatigue as a result of MDR-TB, as it was not confirmed using any formal vestibular assessments. Reports of decreased hearing increased over treatment duration. This concurred with pure tone results showing an increase in hearing thresholds as the duration of the MDR-TB treatment increased.

A prominent high frequency hearing loss from aminoglycoside use was evident, the results of which concur with the study conducted by Jacob *et al*. (2006). With each successive post-treatment assessment, an increased number of participants reported perceptions of hearing loss, with the most common complaint being listening in background noise. This is in keeping with the results of ultra-high frequencies being most affected. Hearing in noisy situations may be significantly impacted when high frequencies are affected (Rademaker-Lakhai *et al*., 2006). Therefore, the need for audiological monitoring programmes at regular intervals to detect early onset ototoxic hearing loss beginning in the high frequencies is highlighted. Early detection of hearing loss and subsequent management may significantly impact an individual’s communication ability, thereby improving quality of life (Fausti *et al*., 2005).

Type A tympanograms were most commonly observed, thereby highlighting adequate functioning of the middle ear system (Fowler & Shanks, 2002) in participants on MDR-TB treatment. Whilst absent ipsilateral acoustic reflex thresholds served to confirm the increasing severity of the hearing loss, reduced sensation levels served to confirm the presence of cochlear hearing loss over treatment duration (Clark, Roeser & Mendrygal, 2007). High frequency acoustic reflex thresholds were more affected and supported the results of pure tone audiometry and DPOAE testing to confirm sloping high frequency sensorineural hearing loss bilaterally.

Pure tone results at baseline revealed normal hearing in 23 participants (44%) on the right ear and 21 participants (40%) on the left ear. Whilst this initial number of participants with normal hearing appears to be low, participants presented with a number of co-morbidities that may contribute to hearing loss. These co-morbid conditions include diabetes mellitus, hypertension, HIV and the use of antiretroviral medication. The exacerbated effects of HIV and TB and the effect of their individual treatments on the auditory system (Harris *et al*., 2012b) must be considered as 49 (94%) of the patients presented with HIV co-infection. Possible changes to the auditory system from use of antiretroviral therapy are an important risk factor to consider; however, such data were not available to the researcher.

Another risk factor for TB and the development of hearing loss is age. Whilst the participants in this study did not fall within the age range for presbycusis, an ageing effect cannot be ruled out as in the case of precocious presbycusis or a response to the medication in older individuals. TB may alter the homeostatic mechanisms of the auditory system in a manner similar to presbycusis, thereby increasing the likelihood of hearing loss in older participants (Brits, Strauss, Eloff, Becker & Swanepoel, 2012). Pre-treatment audiological assessments were therefore used as a baseline to measure hearing function over treatment duration. At baseline, 26 participants (50%) and 28 participants (54%) presented with sensorineural hearing loss on the right and left ears, respectively. As treatment duration increased, so too did the number of participants presenting with sensorineural hearing loss, which increased to 49 (94%) and 50 (96%) participants on the right and left ears, respectively. at post-treatment five. Hearing loss was noted from as early as post-treatment one. These results concur with the study by de Jager and van Altena (2002) who reported the presence of aminoglycoside-induced hearing loss from as early as 5 days. Thus, the need for audiological monitoring, especially when treatment exceeds 3 days, such as in those patients on MDR-TB treatment regimens (Zembower, Nosin, Postelnick, Nguyen & Peterson, 1998), is highlighted. In addition, these results correlate with the available literature suggesting sensorineural nature of hearing loss most common in patients on MDR-TB treatment (Duggal & Sarkar, 2007; Guthrie, 2008; Rademaker-Lakhai *et al*., 2006).

High and ultra-high frequencies were most affected over treatment duration bilaterally. These frequencies progressed from mild to moderate at post-treatment one to severe to profound at post-treatment five. The literature strongly supports high frequency cochlear hearing loss as a result of ototoxicity (Barclay, Kirkpatrick & Begg, 1999; Duggal & Sarkar, 2007; Fausti, Frey, Henry, Olson & Schaffer, 1993; Guthrie, 2008; Jacob *et al*., 2006; Li & Steyger, 2009). Likewise, the steady increases in failed DPOAE results imply increasing severity of hearing loss and cochlear damage over treatment duration (Glattke & Robinette, 2007). The progression of cochlear damage in patients on MDR-TB treatment and subsequent increase in pure tone thresholds has direct implications on an individual’s life. According to Rademaker-Lakhai *et al*. (2006), low-frequency sounds are responsible for the perception of speech in quiet, whereas higher frequency sounds are responsible for perception of speech in noise. Ultra-high frequencies allow for the perception of music as well as appreciation of certain natural sounds (Rademaker-Lakhai *et al*., 2006). Therefore, patients on treatment for MDR-TB may experience increased difficulty with speech discrimination whereby quality of life may be compromised. This is in keeping with results obtained from speech audiometry. Considering the progression of hearing loss over treatment duration, audiological monitoring of patients on MDR-TB treatment is of paramount importance.

Whilst low-frequency deterioration was noted, thresholds still remained essentially normal. The low frequencies have been reported to be least affected as hearing loss in patients on MDR-TB treatment begins in the high frequencies, and with time progresses to the low frequencies (Rademaker-Lakhai *et al*., 2006; Selimoglu, 2007). Therefore, should the study have been conducted over a longer period, progression of hearing loss affecting the low frequencies is anticipated. Progression of hearing loss from mild to profound by post-treatment five reiterates the need for structured audiological monitoring programmes in South Africa, where there is increased aminoglycoside use owing to the incidence of MDR-TB. Whilst hearing loss is the most common side effect of aminoglycoside use, the prevalence of ototoxicity depends on the method used to define hearing loss (Prayle, Watson, Fortnum & Smyth, 2010). Considering the progression of cochlear damage begins with the high frequencies and progresses to the lower frequencies, it is necessary that pure tone audiometry include frequencies higher than those assessed in the conventional testing range of 125 Hz–8 kHz. This highlights the need for appropriate audiological monitoring programmes, including the use of ultra-high frequency audiometry to be implemented in the treatment for MDR-TB. This will allow for early identification of cochlear damage. However, many patients on MDR-TB are too sick or fatigued to cope with the entire test battery; thus, the five-frequency sensitivity range for ototoxicity is recommended (Fausti *et al*., 1993). This was also evident in the current study, where more than half the participants were unable to undergo speech audiometry.

Decreased speech discrimination may also be attributed to the severity of hearing loss (Hodgson, 1980). Patients with speech discrimination difficulties experience feelings of frustration, annoyance, depression and inadequacy, and may withdraw from social activity in an attempt to avoid communication (Hull, 1997). Thus, speech audiometry is a critical component of an audiological monitoring programme. Considering half the sample population were unable to cope with speech audiometry over treatment duration, it is essential that monitoring programmes take this into account and include objective tests such as DPOAE.

The number of overall failed DPOAE results increased over the treatment duration. DPOAEs may serve as a baseline for early detection of ototoxic hearing loss (Glattke & Robinette, 2007). Thus, the marked increase in the number of participants who failed DPOAEs may be attributed to the prolonged exposure to MDR-TB treatment and subsequent hearing loss. In addition, the absence of DPOAEs over treatment duration further highlights cochlear hair cell damage or increasing severity of hearing loss in patients on MDR-TB treatment regimens.

## Conclusion

The participants in this study were tracked over a period of 6 months to detect changes in audiological status following MDR-TB treatment. All participants manifested changes in hearing associated with ototoxicity, using a test battery approach. Changes were noted on pure tone audiometry, speech audiometry, acoustic reflex threshold testing and DPOAEs. The case history reports which allowed for participants to voice their perceptions regarding hearing status confirmed changes in hearing levels and noted other effects such as tinnitus and vertigo. Hearing loss was progressive over time. Deterioration in speech discrimination was an important finding. The study presented a holistic understanding of the effects of ototoxic medication and the need for audiological monitoring and intervention, including counselling, rehabilitation technology and communication intervention. The value of ultra-high frequency audiometry in the detection of ototoxic hearing loss was confirmed.
